# Prospective, randomized, paired-comparison clinical trial of a new universal adhesive in posterior composite restorations: A 2-year evaluation

**DOI:** 10.1007/s00784-026-06799-7

**Published:** 2026-03-09

**Authors:** Jorge Perdigão, Kelli A. Trauger, Carla I. Campbell, Hooi Pin Chew, Ignatius K. Lee, Kacie Woodis

**Affiliations:** 1 Department of Restorative Sciences, Division of Operative Dentistry, University of Minnesota School of Dentistry, Minneapolis, MN 55455 USA; 2 Oral Health Clinical Research Center, University of Minnesota School of Dentistry, Minneapolis, MN 55455 USA; 3 Department of Primary Dental Care, Division of Comprehensive Care, University of Minnesota School of Dentistry, Minneapolis, MN 55455 USA

**Keywords:** Dental bonding, dental adhesion, clinical trial, clinical study

## Abstract

**Objective:**

This prospective, randomized, post-market, paired-comparison controlled trial compared the 2-year clinical performance of a new universal adhesive, Scotchbond Universal Plus Adhesive (SBU+), with that of Scotchbond Universal Adhesive (SBU). Both were applied as self-etch (SE) adhesives for composite resin restoration of Class I and Class II preparations.

**Materials and methods:**

Two posterior teeth in each of 51 subjects were randomized in a 1:1 ratio to a restoration with SBU + or to a restoration with SBU (control) applied with the SE strategy. Class I and/or Class II preparations were restored with Filtek Universal Restorative. Two calibrated and blinded examiners evaluated the restorations at baseline, 6 months, 1 year, and 2 years, using the modified FDI criteria. The Wilcoxon signed-rank test was used to compare the outcomes.

**Results:**

At the 2-year evaluation, retention was 100% for both the SBU + and SBU groups. Regarding fracture and retention, 36 of the 37 SBU+ restorations (97%) were graded “Clinically Excellent/Very Good,” while 1/37 (3%) was graded “Clinically Satisfactory.” All 38 SBU restorations (100%) were graded “Clinically Excellent/Very Good”. Marginal adaptation was clinically acceptable for all restorations at 2 years, with no statistically significant difference between the two adhesive materials. Furthermore, no recurrent caries lesions were observed at 2 years, and no restorations received a “Clinically Insufficient/Unsatisfactory” or “Clinically Poor” grade for any FDI criteria during any evaluation visit.

**Conclusions:**

At 2 years, SBU+ exhibited clinical efficacy similar to its predecessor, SBU, in Class I and Class II restorations in adult patients when using the self-etch (SE) technique.

**Clinical relevance:**

Mildly acidic universal dental adhesives may be used for posterior composite restorations without prior phosphoric acid etching.

## Introduction

Introduced in 2011, universal dental adhesives can be used as etch-and-rinse (ER) or self-etch (SE) adhesives. They can also be used with ‘selective enamel etching’ (SEE), where the adhesive is applied in SE mode on dentin and in ER mode on enamel [1].

Ionic adhesion to tooth structure is a fundamental mechanism for preserving the integrity of the adhesive interface [2, 3]. Universal adhesives contain acidic functional monomers that trigger this ionic bonding to calcium in hydroxyapatite [4, 5]. The functional phosphate monomer 10-methacryloyloxydecyl dihydrogen phosphate (MDP) is commonly used in the composition of universal adhesives, including Scotchbond Universal Plus Adhesive (SBU+, Solventum), and Scotchbond Universal Adhesive (SBU, Solventum). MDP interacts ionically with calcium in dentin and enamel, forming a nano-layered ultrastructure of MDP-Ca salts within the adhesive layer [5–7].

Clinical studies in non-carious cervical lesions (NCCLs) from 2 to 5 years have shown that universal adhesives used with the ER and SEE techniques resulted in higher retention rates compared to the SE technique [8–10]. However, other clinical studies found no difference in retention rates between these adhesion strategies [11–14]. In some clinical trials of Class I and Class II restorations, the retention rate for the SE technique was equivalent to that of the ER and SEE techniques [15–18].

SBU+ offers several advantages over SBU. It is compatible with both dual- and self-cure mechanisms, eliminating the need for a separate dual-cure activator. It also contains a crosslinking radiopaque monomer that is free of Bis-GMA (bisphenol A-glycidyl methacrylate), and its longer silane chain negates the requirement for a separate primer when luting ceramic restorations [19].

Laboratory studies have characterized the adhesion of SBU + to enamel and dentin [20–22] Clinically, SBU+ demonstrated better marginal adaptation than SBU in NCCLs at 24 months with the ER technique [23]. Peer-reviewed clinical trials of SBU + in posterior restorations are scarce. This equivalence study aims to compare the clinical effectiveness of SBU + to SBU (control) in Class I and Class II restorations using the self-etch (SE) technique in adult patients. The null hypothesis is that the 2-year clinical outcomes of SBU+ will not differ from those of SBU when used with the SE technique, as evaluated by the FDI criteria [24].

## Materials and methods

**Fig. 1:**
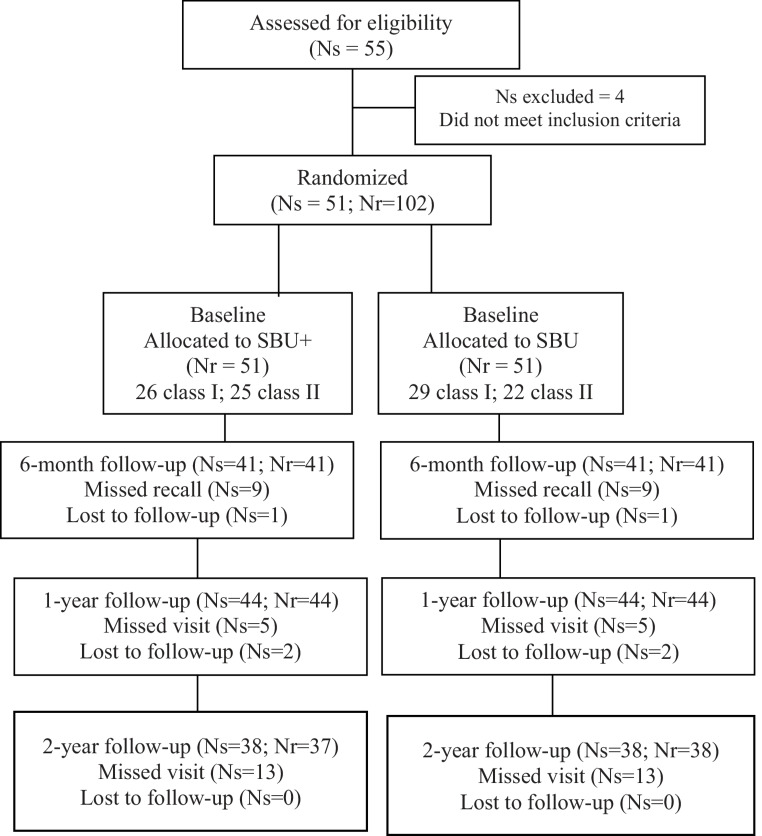
Flow diagram. Ns = Number of subjects. Nr = Number of restorations

This study was approved by the local IRB (Institutional Review Board) and registered at the ClinicalTrials.gov database (number NCT05248204). This prospective, randomized post-market clinical trial followed the Consolidated Standards of Reporting Trials (CONSORT) statements [25, 26] The CONSORT flow diagram is shown in Fig. 1.

### Eligibility criteria

After screening and signing informed consent forms, fifty-one of fifty-five healthy adults were randomized into the study. Inclusion and exclusion criteria are displayed in Table 1. Participants were patients of record at the University of Minnesota School of Dentistry Dental Clinics and were recruited in the order they reported for screening. A calibrated study investigator evaluated subjects for eligibility no more than 21 days before the planned intervention, and eligibility was confirmed on the day of the intervention. Four subjects provided informed consent but did not meet all eligibility criteria at the appointment planned for the interventions. These subjects were ineligible to participate and were considered screening failures.

**Table 1:** Inclusion and exclusion criteria

Inclusion	Exclusion
The Subject is between the ages of 18 and 70 years old at time of consent	The Subject has a history of adverse reaction to any materials used in this study
The Subject requires at least two direct composite restorations for posterior Class I or Class II carious lesions in vital teeth that are asymptomatic and with minimal mobility (< 1 mm in the buccolingual direction)	The Subject is pregnant or breast feeding at the time of screening
Carious lesions have a minimum buccolingual diameter of the occlusal surface that is at least 1/3 of the distance between the buccal and lingual cusps	The Subject has fewer than 20 teeth
Teeth selected for Class II restorations need to have an adjacent tooth in a position that will allow for a contact relationship to be established	The Subject is taking part in or planned to be enrolled in an evaluation of other restorative materials at any time during the study
The Subject is able and willing to sign Informed Consent Form in English without assistance	The Subject has advanced periodontal disease (i.e., Grace and Smales Mobility Index ≥ 2) that involve the study teeth
The Subject is able and willing to be available for all scheduled study visits	The Subject had orthodontic appliance treatment within the previous 3 months
The Subject is in good general health (i.e., meets ASA* Level I or ASA Level II physical status classification criteria)	The Subject has pronounced enamel wear facets, indicting severe, on-going bruxism
The selected teeth need to have occlusal contact with an antagonistic natural tooth	The Subject has severe xerostomia
The Subject has existing radiographic images of the study teeth of current and acceptable diagnostic quality obtained within the previous 12 months	The study tooth has a history of or existing, prolonged tooth hypersensitivity
	Pulp exposure of either study tooth during restoration procedure
	The study tooth is an abutment for fixed or removable prostheses
	The study tooth has a fracture or is visibly cracked such that it may impact the longevity of the tooth
	The carious lesion is stage RC5 or RC6 of the ICDAS** radiographic scoring system
	The Subject is unable to understand study procedures or provide consent in English
	The Subject is an employee or student of the study investigator(s)

*ASA American Society of Anesthesiologists (https://www.asahq.org/standards-and-practice-parameters/statement-on-asa-physical-status-classification-system), **ICDAS International Caries Detection and Assessment System (https://www.iccms-web.com/uploads/asset/5928404ea4df6343406124.pdf

### Baseline demographic characteristics

Subject demographics are summarized in Table 2. Of the 51 subjects allocated to treatment in this study, 55% were female and 45% were male, with ages ranging from 18 to 68 years (median age, 30 years). Each subject received both treatments and served as their own control.

**Table 2:** Summary of Study Participant Demographics

	Treated subjects
Gender	
Male	23 (45%)
Female	28 (55%)
Age at time of consent (years)	
Range	18–68
Mean (SD)	33 (12)
Median	30

### Trial design, settings and location of data collection

This prospective, double-blind, randomized, paired-comparison clinical trial compared the clinical efficacy of SBU+ with that of its predecessor, SBU (Table 3), for restoring Class I and/or Class II preparations in adult patients using the SE mode. Restorations were placed and evaluated at baseline, 6 months, 1 year, and 2 years at the University of Minnesota Oral Health Clinical Research Center between June 2022 and March 2025.

**Table 3:** Materials, compositions^19^ and application mode

Adhesive	Composition	Application
Scotchbond Universal Plus Adhesive (Solventum)	MDP, radiopaque monomer, HEMA, filler, (3-methacryloyloxypropyl) triethoxysilane, APTES, ethanol, water, camphorquinone, copolymer of acrylic and itaconic acid, copper (II) acetate monohydrate	-Apply the adhesive with agitation for 20 s -Air dry for at least 5 s until adhesive does not move anymore. -Light cure for 10 s.
Scotchbond Universal Adhesive (Solventum)	MDP, phosphate monomer, Bis-GMA, HEMA, copolymer of acrylic and itaconic acid, filler, ethanol, water, camphorquinone, silane.	-Apply the adhesive to the prepared tooth and rub it in for 20 s -Gently air dry the adhesive for approximately 5 s to evaporate the solvent -Light cure for 10 s.

APTES: (3-Aminopropyl) triethoxysilane; Bis-GMA: bisphenol a diglycidyl ether dimethacrylate; HEMA- 2-hydroxyethyl methacrylate

### Sample size

Several authors have reported an annual failure rate of posterior composite restorations ranging from ≈ 1.0–3.5% [27–30] We estimated that a sample size of 50 subjects accounted for 20% attrition to allow a minimum of 40 subjects to complete the study at 2 years. In addition, the sample size of 100 restorations (2 restorations per subject) would provide adequate accuracy in estimating the overall restoration survival rate. For an expected annual restoration failure rate of 3.3%, a one-sided 95% confidence interval would generate an upper confidence limit of 7.7%, using the Wilson score method.

### Randomization and allocation concealment

Upon enrollment, each subject was assigned a unique identification number. All study data were reported using this unique subject identifier, which contained no information that could identify the subject. Randomization was performed after tooth preparation for restoration but before the application of the adhesive. The premolars and molars were numbered using the Universal Numbering System (premolars = 4–5, 12–13, 20–21, or 28–29; molars = 1–3, 14–16, 17–19, or 30–32) and the randomization of these teeth to study treatments were centralized and carried out electronically (Zelta, Merative, Ann Arbor, MI, USA) by the study coordinator.

The two posterior teeth from each subject were randomized in a 1:1 ratio, with one tooth receiving a restoration using SBU + and the other receiving a restoration using SBU (control). Study teeth were assigned randomization numbers, beginning with the tooth having the lowest number. The first adhesive in the randomization schedule was then assigned to the tooth with the lower number in each pair.

### Blinding

For all evaluations, both the subjects and the evaluators were blinded to group allocation. The five operators who performed the interventions were not blinded to the interventions because the individual doses of the two adhesives were different colors.

### Outcomes

The FDI World Dental Federation criteria [24] were used to assess the esthetic, functional, and biological properties of the restorations. Table 4 shows the specific primary and secondary endpoints assessed in this study.

**Table 4:** – Primary and secondary endpoints

Primary	Secondary
Proportion of subjects with partial or complete loss of the restoration	Time to restoration failure (i.e., time to partial or complete loss of restoration)
Proportion of subjects with fracture of the restorative material	Proximal anatomical form (contact point)
Marginal adaptation	Incidence of recurrent caries
	Marginal staining
	Surface staining
	Color match and translucency
	Surface luster
	Postoperative sensitivity

### Interventions

Each of the 51 subjects had two teeth restored, resulting in 102 treated teeth. Restorations were placed sequentially following the manufacturer’s instructions for use (Table 3). All operators had at least two years of clinical experience using the same materials. Prior to restoration placement, the study director reviewed the instructions for use with all other four clinical investigators.

Local anesthesia was administered to all subjects. The caries lesion or existing restoration was accessed using a high-speed handpiece under water irrigation. When present, carious dentin was removed to the level of affected dentin using a slow-speed tungsten round bur. Following tooth preparation, isolation was achieved with Isovac (Zyris, Inc., Santa Barbara, CA). For Class II restorations, a V3-ring sectional matrix and corresponding wedge (Triodent, Katikati, New Zealand) were adapted to the proximal box. In preparations considered to be in deep dentin, a minimal amount of hard-setting calcium hydroxide (Life, Kerr Dental, Brea, CA) was applied. A type III resin-modified glass ionomer cement (3M Vitrebond Plus, Solventum) was then placed over the calcium hydroxide liner and light-cured for 20 s using a new, calibrated 3M Elipar DeepCure-S LED Curing Light (Solventum). According to the manufacturer, the irradiance of this curing light is 1470 mW/cm^2^ (-10%/+20%). The irradiance for the same unit has been reported as 1419 mW/cm^2^ in peer-reviewed literature [31].

Either SBU + or SBU were applied into the preparations as SE adhesives following the manufacturer’s directions (Table 3). After polymerizing the adhesive with a calibrated Elipar DeepCure-S LED Curing Light (Solventum) and selecting the composite resin shade, the restorative material 3 M Filtek Universal Restorative (Solventum) was placed in 2 mm increments, with each increment light-cured for 10 s using the same curing light. Following verification and adjustment of the bite, the restorations were finished and polished using 3 M Sof-Lex disks and the 3 M Sof-Lex Diamond Polishing System (Solventum).

Table 5 summarizes the characteristics of the preparation and treatment groups.

**Table 5:** Summary of tooth and preparation characteristics by treatment arm

	Scotchbond Universal Plus Adhesive (SBU+) (*N* = 51)	Scotchbond Universal Adhesive (SBU) (*N* = 51)	Total (*N* = 102)	
Tooth preparation				
Class I	26 (51%) Primary caries lesion – 24 Restoration replacement − 2	29 (57%) Primary caries lesion – 23 Restoration replacement − 6	55 (54%)	
Class II	25 (49%) *Two surfaces − 20* Primary caries lesion − 18 Restoration replacement − 2 *Three surfaces – 5* Primary caries lesion − 4 Restoration replacement − 1	22 (43%) *Two surfaces − 19* Primary caries lesion − 17 Restoration replacement − 2 *Three surfaces − 3* Primary caries lesion − 3 Restoration replacement − 0	47 (46%)	
Indirect pulp capping performed				
Yes	5 (10%)	6 (12%)	11 (11%)	
No	46 (90%)	45 (88%)	91 (89%)	
Tooth				
Premolar	20 (39%)	10 (20%)	30 (29%)	
Molar	31 (61%)	41 (80%)	72 (71%)	
Location				
Maxillary	23 (45%)	27 (53%)	50 (49%)	
Mandibular	28 (55%)	24 (47%)	52 (51%)	

### Evaluation

All assessments were performed by two clinical investigators from the study team, neither of whom placed the restorations. These examiners were blinded to treatment allocation and conducted their evaluations independently of each other. After both examiners completed their assessments, their scores were compared for each criterion at each visit, and discrepancies were resolved by consensus before the subject left the clinic. Prior to subject assessment, all examiners underwent training and calibration exercises for the FDI scoring criteria [24] (Table 6). Training and calibration for the FDI criteria were conducted three weeks prior to the commencement of the study, with the exception of the recurrent caries criterion, which was conducted three weeks before the six-month follow-up visit. The training exercise utilized a set of 13 photographs depicting Class I and II composite restorations representing the full range of possible scores. Calibration was performed using a separate set of 25 photographs of Class I and II composite restorations, also representing the full range of scores. Inter-rater and intra-rater agreement measures were obtained through two calibration exercises conducted two weeks apart. The inter-rater agreement was analyzed with the Cohen Weighted Kappa statistics using SPSS Statistics 27 (IBM, Armonk, New York). The mean inter-rater Cohen’s Kappa coefficient ranged from 0.63 to 0.87 for all FDI criteria assessed in this study except for color match and translucency, for which the Cohen’s Kappa coefficient was 0.55. Examiners were recalibrated after the 6-month visit when a new evaluator was added to the study team.

**Table 6:** FDI criteria evaluated in this study^24^

	Functional properties				Esthetic properties				Biological properties		
	Fractures and retention	Marginal adaptation		Proximal contact	Marginal staining a. Surface; b. Margin	Color match and translucency		Surface luster	Postoperative (hyper-) sensitivity		Recurrence of caries
1. Clinically excellent/very good	1. No fractures / cracks	1. Harmonious outline, no gaps, no white or discolored lines		1.1. Normal contact point (floss or 25 μm metal blade can pass) 1.2 Normal contour	1a1. No surface staining 1b1. No marginal staining	1. Good color match, no difference in shade and/or translucency		1. Luster comparable to enamel.	1. No hypersensitivity, normal vitality		1. No secondary or primary caries
2. Clinically good	2. Small hairline crack.	2.1. Marginal gap (150 μm), white lines 2.2. Small marginal fracture removable by polishing. 2.3. Slight ditching, slight step/flashes, minor irregularities		2.1. Contact slightly too strong but no disadvantage (floss or 25 μm metal blade can only pass with pressure) 2.2. Slightly deficient contour	2a1. Minor surface staining, easily removed by polishing 2b1. Minor marginal staining, easily removable by polishing.	2. Minor deviations in shade and/or translucency		2.1 Slightly dull, not noticeable from speaking distance 2.2 Some isolated pores	2. Minor hypersensitivity for a limited period of time, normal vitality		2 Small and localized a. demineralization. b. erosion c. abfraction
3.Clinically sufficient / satisfactory (minor shortcomings with no unacceptable effects but not adjustable without damage)	3. Two or more or larger hairline cracks and/or material chip fracture not affecting the marginal integrity or proximal contact	3.1. Gap < 250 μm not removable 3.2. Several small marginal fractures 3.3. Major irregularities, ditching or flash, steps		3.1. Somewhat weak contact, no indication of damage to tooth, gingiva or periodontal structures; 25 μm metal blade can pass 3.2. Visibly deficient contour	3a1. Moderate surface staining that may also present on other teeth, not esthetically unacceptable 3b1. Moderate marginal staining, not esthetically unacceptable	3. Distinct deviation but acceptable. Does not affect esthetics		3.1. Dull surface but acceptable if covered with film of saliva 3.2 Multiple pores on more than one third of the surface	3.1. Moderate hypersensitivity 3.2. Delayed/mild sensitivity; no subjective complaints, no treatment needed.		3.Larger areas of demineralization, dentine not exposed. Only preventive measures necessary
4. Clinically unsatisfactory (but repairable)	4.1. Material chip fractures which damage marginal quality or proximal contacts 4.2. Bulk fractures with partial loss (less than half of the restoration)	4.1. Gap > 250 μm or dentine/base exposed. 4.2. Severe ditching or marginal fractures 4.3. Large irregularities or steps (repair necessary)		4.1. Too weak and possible damage due to food impaction; 100 μm metal blade can pass 4.2. Inadequate contour. Repair possible	4a1. Unacceptable surface staining on the restoration and major intervention necessary for improvement 4b1. Pronounced marginal staining, major intervention necessary for improvement	4. Localized clinical deviation that can be corrected by repair		4.1. Rough surface cannot be masked by saliva film, simple polishing is not sufficient. Further intervention necessary 4.2. Voids	4.1. Intense hypersensitivity 4.2. Delayed with minor subjective symptoms 4.3. No clinical detectable sensitivity. Intervention necessary but not replacement.		4.1. Caries with cavitation and suspected undermining caries 4.2. Erosion in dentin 4.3. Abrasion/abfraction in dentin (for class V restorations). Localized and accessible, can be repaired
5. Clinically poor (replacement necessary)	5. (Partial or complete) loss of restoration or multiple fractures.	5.1. Restoration (complete or partial) is loose but in situ 5.2. Generalized major gaps or irregularities	5.1. Too weak and/or clear damage due to food impaction and/or pain/gingivitis 5.2. Insufficient contour, requires replacement		5a1. Severe surface staining and/or subsurface staining, generalized or localized, not accessible for intervention 5b1. Deep marginal staining, not accessible for intervention	5. Unacceptable. Replacement necessary.	5. Very rough, unacceptable plaque retentive surface		5. Intense, acute pulpitis or nonvital tooth. Endodontic treatment is necessary, and restoration has to be replaced.	5. Deep secondary caries or exposed dentine that is not accessible for repair of restoration.	

### Statistics

All analyses were conducted using SAS v9.4 software (SAS, Cary, NC, USA). Statistical tests were two-tailed and performed with a significance level of 5%. The corresponding p-values and 95% confidence intervals were reported for all analyses.

This was a paired-comparison design study. The analysis of all endpoints was based on all randomized subjects who had post-baseline data available.

### Analysis of primary and secondary endpoints

Primary endpoints were analyzed with and without imputation; the results were similar in both analysis sets. Secondary endpoints were analyzed using the per-protocol approach. Because the secondary endpoints were more exploratory and not decision-critical, a unimputed analysis was deemed reasonable to avoid model-dependent assumptions and excess analysis burden.

Descriptive statistics summarized the primary endpoints overall and for each adhesive product at baseline, 6 months, 1 year, and 2 years. The Wilcoxon signed-rank test and McNemar (or Cochran-Mantel-Haenszel) test compared the criteria outcomes. Descriptive statistics also summarized the secondary endpoints, and the Wilcoxon signed-rank test compared outcomes at each time point. Time to restoration failure was not analyzed due to the low failure rate.

## Results

At the 6-month follow-up, 10 subjects missed their visit (one early withdrawal, nine still enrolled). Seven subjects missed the 1-year visit (two early withdrawals, five still enrolled). By the 2-year visit, 13 subjects had withdrawn from the study. Additionally, one subject had one tooth withdrawn at the 2-year visit due to non-compliance with the study protocol. (Fig. 1)

The gradings for the FDI criteria assessed in this clinical trial are displayed in Table 7. No restorations were graded as “Clinically Insufficient/Unsatisfactory” or “Clinically Poor” for any of the FDI criteria at the baseline, 6-month, 1-year, or 2-year visits. Differences in FDI criteria between the groups did not reach statistical significance at baseline, 1 year, and 2 years.

**Table 7:** FDI gradings for each of the criteria at baseline, 6 months, 1 year and 2 years

	FDI Criteria	FDI gradings	Baseline			6 months			1 year		2 years		
			SBU+ (*N* = 51)	SBU (*N* = 51)	SBU+ (*N* = 41)		SBU (*N* = 41)	SBU+ (*N* = 44)		SBU (*N* = 44)		SBU+ (*N* = 37)	SBU (*N* = 38)
Functional Properties	Retention of Restorative Material	EV	51 (100%)	51 (100%)	41 (100%)		41 (100%)	44 (100%)		44 (100%)		36 (97%)	38 (100%)
		GD	0 (0%)	0 (0%)	0 (0%)		0 (0%)	0 (0%)		0 (0%)		1 (3%)	0 (0%)
		SA	0 (0%)	0 (0%)	0 (0%)		0 (0%)	0 (0%)		0 (0%)		0 (0%)	0 (0%)
		US/PO	0 (0%)	0 (0%)	0 (0%)		0 (0%)	0 (0%)		0 (0%)		0 (0%)	0 (0%)
	Fracture of Restorative Material	EV	51 (100%)	51 (100%)	38 (93%)		40 (98%)	42 (95%)		44 (100%		36 (97%)	38 (100%)
		GD	0 (0%)	0 (0%)	1 (2%)		1 (2%)	1 (2%)		0 (0%)		0 (0%)	0 (0%)
		SA	0 (0%)	0 (0%)	2 (5%)		0 (0%)	1 (2%)		0 (0%)		1 (3%)	0 (0%)
		US/PO	0 (0%)	0 (0%)	0 (0%)		0 (0%)	0 (0%)		0 (0%)		0 (0%)	0 (0%)
	Marginal adaptation	EV	17 (33%)	19 (37%)	9 (22%)		7 (17%)	9 (20%)		13 (30%)		3 (8%)	2 (5%)
		GD	34 (67%)	32 (63%)	31 (76%)		33 (80%)	35 (80%)		29 (66%)		34 (92%)	35 (92%)
		SA	0 (0%)	0 (0%)	1 (2%)		1 (2%)	0 (0%)		2 (5%)		0 (0%)	1 (3%)
		US/PO	0 (0%)	0 (0%)	0 (0%)		0 (0%)	0 (0%)		0 (0%)		0 (0%)	0 (0%)
	Proximal contact^#^	EV	23 (92%)	22 (100%)	17 (81%)		18 (95%)	17 (74%)		19 (100%)		14 (82%)	18 (100%)
		GD	1 (4%)	0 (0%)	1 (5%)		0 (0%)	1 (4%)		0 (0%)		3 (18%)	0 (0%)
		SA	1 (4%)	0 (0%)	3 (14%)		1 (5%	2 (9%)		0 (0%)		0 (0%)	0 (0%)
		US/PO	0 (0%)	0 (0%)	0 (0%)		0 (0%)	0 (0%)		0 (0%)		0 (0%)	0 (0%)
Esthetic Properties	Surface Luster (Polish Retention/Surface Gloss/ Roughness	EV	44 (86%)	43 (84%)	18 (44%)		25 (61%)	31 (70%)		33 (75%)		30 (81%)	32 (84%)
		GD	7 (14%)	8 (16%)	22 (54%)		15 (37%)	12 (27%		11 (25%)		7 (19%)	6 (16%)
		SA	0 (0%)	0 (0%)	1 (2%)		1 (2%)	1 (2%)		0 (0%)		0 (0%)	0 (0%)
		US/PO	0 (0%)	0 (0%)	0 (0%)		0 (0%)	0 (0%)		0 (0%)		0 (0%)	0 (0%)
	Surface staining	EV	50 (98%)	50 (98%)	41 (100%)		41 (100%)	39 (89%)		44 (100%)		35 (95%)	37 (97%)
		GD	1 (2%)	1 (2%)	0 (0%)		0 (0%)	4 (9%)		0 (0%)		2 (5%)	1 (3%)
		SA	0 (0%)	0 (0%)	0 (0%)		0 (0%)	1 (2%)		0 (0%)		0 (0%)	0 (0%)
		US/PO	0 (0%)	0 (0%)	0 (0%)		0 (0%)	0 (0%)		0 (0%)		0 (0%)	0 (0%)
	Marginal staining	EV	49 (96%)	49 (96%)	37 (90%)		36 (88%)	39 (89%)		39 (89%)		33 (89%)	31 (82%)
		GD	2 (4%)	2 (4%)	3 (7%)		4 (10%)	4 (9%)		5 (11%)		2 (5%)	7 (18%)
		SA	0 (0%)	0 (0%)	1 (2%)		1 (2%)	1 (2%)		0 (0%)		2 (5%)	0 (0%)
		US/PO	0 (0%)	0 (0%)	0 (0%)		0 (0%)	0 (0%)		0 (0%)		0 (0%)	0 (0%)
	Color match and translucency	EV	37 (73%)	35 (69%)	30 (73%)		19 (46%)	33 (75%)		35 (80%)		25 (68%)	22 (58%)
		GD	13 (25%)	15 (29%)	11 (27%)		21 (51%)	11 (25%)		9 (20%)		12 (32%)	16 (42%
		SA	1 (2%)	1 (2%)	0 (0%)		1 (2%)	0 (0%)		0 (0%)		0 (0%)	0 (0%)
		US/PO	0 (0%)	0 (0%)	0 (0%)		0 (0%)	0 (0%)		0 (0%)		0 (0%)	0 (0%)
Biological Properties	Postoperative Hypersensitivity and Tooth Vitality	EV	51 (100%)	51 (100%)	41 (100%)		41 (100%)	44 (100%)		42 (95%)		37 (100%)	38 (100%)
		GD	0 (0%)	0 (0%)	0 (0%)		0 (0%)	0 (0%)		2 (5%)		0 (0%)	0 (0%)
		SA	0 (0%)	0 (0%)	0 (0%)		0 (0%)	0 (0%)		0 (0%)		0 (0%)	0 (0%)
		US/PO	0 (0%)	0 (0%)	0 (0%)		0 (0%)	0 (0%)		0 (0%)		0 (0%)	0 (0%)
	Recurrent caries	EV	51 (100%)	51 (100%)	41 (100%)		41 (100%)	44 (100%)		44 (100%)		37 (100%)	38 (100%)
		GD	0 (0%)	0 (0%)	0 (0%)		0 (0%)	0 (0%)		0 (0%)		0 (0%)	0 (0%)
		SA	0 (0%)	0 (0%)	0 (0%)		0 (0%)	0 (0%)		0 (0%)		0 (0%)	0 (0%)
		US/PO	0 (0%)	0 (0%)	0 (0%)		0 (0%)	0 (0%)		0 (0%)		0 (0%)	0 (0%)

* - *EV *clinically excellent/very good, *GD* clinically good, *SA* clinically sufficient/satisfactory, *US/PO* clinically unsatisfactory/clinically poor

# - Not applicable to class I restorations: 26 SBU + and 29 SBU at baseline; 18 SBU + and 20 SBU at 2 years. Two teeth with an SBU+ Class II restoration were adjacent to a tooth that was extracted after restoration of the study tooth

At 6 months, both treatment groups showed some reduction in properties such as marginal adaptation, marginal staining, and surface luster compared to baseline, although all were still graded as clinically acceptable. There were no significant differences between the groups in scores for fracture of restorative materials, marginal adaptation, marginal staining, surface luster, and postoperative sensitivity. The results for color match and translucency were lower in the SBU group compared to the SBU+ group (*p* = 0.016). All restorations were graded “Clinically Excellent/Very Good” (absence of postoperative sensitivity) for postoperative sensitivity at 6 months.

At 1 year, there were no restoration failures for the primary endpoints (Table 7). The retention was 100% for both SBU + and SBU. For the FDI criterion fracture and retention (Table 6) 42/44 SBU+ restorations (95%) and 44/44 SBU restorations (100%) were graded “Clinically Excellent/Very Good” (*p* > 0.05). One SBU+ (2%) restoration was graded “Clinically Good” and one SBU+ restoration (2%) was graded “Clinically Satisfactory”. All restorations were clinically acceptable for marginal adaptation at 1 year with no statistical differences between the two adhesive materials (*p* > 0.05). Twenty-two restorations (9 SBU + and 13 SBU) scored as “Clinically Excellent/Very Good”, 64 restorations scored as “Clinically Good” (35 SBU + and 29 SBU), and two SBU restorations scored as “Clinically Satisfactory”.

At 2 years, the retention was 100% for both SBU + and SBU. For the FDI criterion fracture and retention, 36 of the 37 SBU+ restorations were graded “Clinically Excellent/Very Good” (97%), and 1 of the 37 SBU+ restorations was graded “Clinically Satisfactory” (3%) at 2 years. In the SBU group, all 38 restorations (100%) were graded “Clinically Excellent/Very Good” (*p* > 0.05). All restorations demonstrated clinically acceptable marginal adaptation at 2 years, with no statistically significant difference between the two adhesive materials. Furthermore, no recurrent caries lesions were observed at the 2-year follow-up.

At 2 years, proximal anatomical form, a secondary endpoint, was only scored for Class II restorations. For two subjects, the tooth adjacent to the treated study tooth was extracted before the 1-year visit. Consequently, proximal anatomical form was marked as “N/A” (not applicable) for these two teeth and all Class I restorations. The remaining Class II restorations were graded at least “Clinically Good” for proximal contacts. Although the SBU group showed slightly better proximal anatomical form, this difference was not statistically significant (*p* > 0.05). For postoperative sensitivity, 100% of both SBU+ (37/37) and SBU (38/38) restorations were graded “Clinically Excellent/Very Good,” indicating an absence of postoperative sensitivity. Surface staining was graded “Clinically Excellent/Very Good” for 95% (35/37) of SBU+ restorations and 97% (37/38) of SBU restorations (*p* > 0.05). While the SBU group had significantly lower scores for color match and translucency at 6 months compared to the SBU+ group, no statistically significant difference was observed at 1 year and 2 years (*p* > 0.05). All other secondary endpoints were scored as “Clinically Excellent/Very Good” or “Clinically Good.”

## Discussion

The release of dentin calcium caused by the mild acidity of SBU + and SBU (pH = 2.7) promotes the formation of stable MDP-Ca salts, leading to stable ionic bonds and increased bond strength [3, 4, 32, 33]. While the etching potential of mild SE adhesives on enamel is not as strong as that of phosphoric acid, the MDP included in SBU (10–20%) [34] and SBU+ (< 20%) [35] exceeds the etching potential of other acidic functional monomers used in dentin adhesives. This slight etching potential of MDP, along with its ability to bond ionically to calcium, may explain the identical clinical effectiveness of SBU when applied as either an etch-and-rinse (ER) or SE adhesive in non-carious cervical lesions (NCCLs) after 5 years [36]. The ionic bonding provided by MDP may also account for the statistically similar 2-year clinical outcomes in NCCLs when using SBU in SE mode compared to Clearfil SE Bond, a two-step SE adhesive considered a gold standard [37].

SBU has demonstrated excellent retention in Class I and Class II restorations when used as a SE adhesive [15–18], even when compared to the ER strategy [16]. A recent clinical trial [38] reported that the outcomes of posterior composite restorations with SBU applied with the SE, ER and SEE modes were similar at 26 months, except for marginal discoloration for which the ER mode resulted in statistically better scores than the SE mode. A study of posterior composite restorations at 3 years [39] concluded that SBU applied in SEE and ER modes resulted in better clinical performance than the SE mode in terms of restoration survival and the formation of marginal gaps, with SEE reaching the best outcomes. In another clinical study the clinical efficacy of the SBU + in posterior restorations was similar to that of SBU at 1 year in SE mode [40].

In contrast, universal adhesives lacking MDP have shown limited clinical success. For example, a randomized clinical trial reported a 34% loss rate at 3 years for restorations placed in NCCLs using an MDP-free universal adhesive. The retention rate for restorations placed using the self-etch technique was also notably low (48.4%) [41]. Similarly, another clinical trial found statistically significant higher marginal discoloration in NCCLs at 5 years with an MDP-free universal adhesive compared to SBU [36].

In the ER technique, phosphoric acid can decalcify dentin up to 5 μm, leaving collagen fibers without hydroxyapatite [42] and potentially hindering the ionic bonding of MDP-containing adhesives. However, SBU used in ER has demonstrated excellent restoration retention rates in non-carious cervical lesions (NCCLs) after 5 years [8]. In addition to the ionic bonding provided by phosphate monomers like MDP, polycarboxylate monomers have also demonstrated ionic adhesion to dentin [43]. For example, SBU + and SBU contain a copolymer of acrylic and itaconic acids, also known as polyalkenoic acid copolymer or 3M Vitrebond copolymer [44] used for the first time in a resin-modified glass-ionomer cement (RMGIC) [45]. Nuclear magnetic resonance (NMR) spectroscopy has confirmed ionic adhesion between this polyalkenoic acid copolymer and hydroxyapatite for SBU and two other dental adhesives, detecting adhesion between the polyalkenoic acid copolymer in SBU and hydroxyapatite [46]. Specifically, carboxyl groups within polyalkenoic acids replace phosphate ions in hydroxyapatite, establishing ionic bonding with calcium [43, 44, 47].

The polyalkenoic acid copolymer may have positively affected the clinical behavior of both SBU + and SBU in our study, which aligns with other clinical studies reporting excellent retention for dental adhesives containing this copolymer (48,49]. Furthermore, a systematic review concluded that GIC-based materials resulted in the lowest annual failure rate in NCCLs (2.0%) across all adhesive materials, including 2-step SE and 3-step ER adhesives [50]. These findings underscore the significant role of polyalkenoic polymers in providing reliable ionic bonding to calcium in mineralized tooth substrates.

One limitation of this clinical study is the use of two universal adhesives with similar compositions, applied using an adhesive strategy considered less effective on enamel than the ER strategy. Nevertheless, the outcomes of this randomized clinical trial, along with those of a previous clinical trial using self-etch bonding (SBU) [16], may challenge this concept and highlight the potential role of ionic bonding to mineralized tooth structure. A second potential limitation was the recall rate of approximately 75% at 2 years. A larger-than-expected attrition rate was observed (25.4% versus the predicted 20%), resulting in fewer subjects with primary 2-year data than planned (38 instead of 40). A third limitation is that this clinical trial was carried out in an academic clinical research center, which may not mirror the clinical setting of a private dental practice.

We failed to reject the null hypothesis, as the 2-year clinical outcomes of SBU and SBU+ used in self-etch mode were not significantly different based on the FDI criteria.

## Conclusion

At 2 years, Scotchbond Universal Plus Adhesive demonstrated clinical efficacy statistically equivalent to Scotchbond Universal Adhesive in posterior composite restorations of adult patients using the SE adhesion strategy. There were no restoration failures or recurrent caries lesions for either adhesive. All FDI criteria were clinically acceptable for both adhesives. 

## Data Availability

Regarding the availability of the data, datasets generated or analyzed during the study are property of the sponsor.
